# CT Anatomy of the Skull in Lamb and Adult Romane Sheep

**DOI:** 10.1155/vmi/5517229

**Published:** 2026-08-12

**Authors:** Omid Zehtabvar, Majid Masoudifard, Javad Abbasi, Javid Sepahtash, Fateme Pariz, Seyyed Hossein Modarres Tonekabony

**Affiliations:** ^1^ Anatomy sector, Department of Basic Science, Faculty of Veterinary Medicine, University of Tehran, Tehran, Iran, ut.ac.ir; ^2^ Department of Surgery and Radiology, Faculty of Veterinary Medicine, University of Tehran, Tehran, Iran, ut.ac.ir; ^3^ Department of Animal and Poultry Health and Nutrition, Faculty of Veterinary Medicine, University of Tehran, Tehran, Iran, ut.ac.ir; ^4^ DVM Student, Faculty of Veterinary Medicine, University of Tehran, Tehran, Iran, ut.ac.ir; ^5^ Department of Food Hygiene and Quality Control, Faculty of Veterinary Medicine, University of Tehran, Tehran, Iran, ut.ac.ir

**Keywords:** anatomy, CT scan, morphology, Romane sheep, volumetry

## Abstract

This study investigates the cranial computed tomography (CT) anatomy of Romane sheep, a composite breed derived from Romanov and Berrichon du Cher sheep, known for its adaptability to temperate climates and desirable genetic traits such as high fertility and robustness. The heads of three lambs and five adult sheep, sourced from a Tehran farm, were examined using a Siemens Somatom Spirit 2 CT scanner. Transverse, sagittal, and dorsal CT images were analyzed using RadiAnt and Marcopacs software, while morphometric and volumetric parameters, including skull dimensions, mandibular and hyoid bone measurements, teeth lengths, and sinus volumes, were evaluated. Lambs exhibited compact cranial structures, with an average skull length of 13.80 cm, head volume of 340.93 cm^3^, and smaller sinus volumes. Conversely, adult Romane sheep displayed significant growth, with skull length doubling to 23.16 cm, head volume increasing to 615 cm^3^, and expanded sinus volumes. These findings demonstrate substantial cranial development as lambs mature. The study emphasizes the importance of defining normal cranial anatomy for Romane sheep. Detailed anatomical data are crucial for diagnosing abnormalities, surgical planning, and identifying breed‐specific variations. This research provides a reference for comparative studies. Overall, the results highlight the vital role of advanced anatomical studies in advancing veterinary medicine, genetic research, and livestock management.

## 1. Introduction

The Romane sheep breed, a composite breed resulting from the crossing of Romanov and Berrichon du Cher sheep, is notable for its adaptability to various temperate breeding conditions, both intensive and extensive [1]. This breed has been the subject of various genetic studies aimed at understanding its unique traits, particularly in relation to body condition and wool characteristics. For instance, Macé et al. conducted a genome‐wide association study (GWAS) that identified significant genetic variants associated with body fat reserves in ewes, highlighting the genetic architecture that underpins body condition traits in Romane sheep [2].

Computed tomography (CT) has emerged as a vital imaging modality in veterinary medicine, particularly for the detailed examination of skull anatomy in sheep. The application of CT scans allows for enhanced visualization of both bone and soft tissue structures, which is essential for understanding the complex anatomy of the sheep skull. This imaging technique is particularly beneficial for differentiating between various anatomical features, such as the nasal cavity and paranasal sinuses, which are critical for both clinical assessments and research purposes [3].

The CT anatomy of the sheep skull has been extensively studied, revealing significant insights into its structure and the relationships between various cranial components. CT imaging provides high‐resolution images that facilitate the differentiation between bone and soft tissue, which is crucial for understanding the complex anatomy of the sheep skull, including the nasal and paranasal sinuses, cranial cavities, and overall skull morphology [4, 5].

One of the key studies in this area is by Awaad et al., which utilized CT to explore the surgical anatomy of the skull sinuses in Egyptian native sheep. Their findings demonstrated that CT scans allowed for better discrimination between the nasal passages and paranasal sinuses, highlighting the utility of this imaging technique in veterinary anatomy [6]. Similarly, Masoudifard et al. conducted a detailed examination of the head anatomy in Ile de France sheep using CT, confirming the extension of the maxillary sinus and its anatomical relationships with surrounding structures, which aligns with findings from other studies on small ruminants [3]. This consistency across studies underscores the reliability of CT in elucidating the anatomical features of sheep skulls.

Moreover, the morphometric analysis of sheep skulls has revealed similarities to human cranial anatomy, particularly in terms of skull thickness and curvature. Trovatelli et al. noted that the neuroanatomical features of sheep exhibit overlapping characteristics with humans, which can be beneficial for translational research [7]. This is further supported by the work of Banstola and Reynolds, who emphasized the anatomical parallels between sheep and human brains, particularly in subcortical structures [8]. Such comparisons are vital for understanding the implications of ovine models in human medical research.

CT imaging has also proven invaluable in the assessment of the paranasal sinuses, which are critical for understanding respiratory health in sheep. The study by Macias‐Valle et al. provided a comprehensive evaluation of sinonasal anatomy using CT, establishing a foundation for future rhinologic research [9]. Nasal and paranasal sinuses have also been investigated in Shal sheep [10]. Additionally, the work of Hoffmann et al. on the cerebral venous system in sheep illustrates the broader applications of CT in studying cranial anatomy and its relevance to experimental models [11].

In conclusion, the CT anatomy of the skull in Romane sheep represents a vital area of research that bridges veterinary and human medicine. The detailed imaging capabilities provided by CT technology facilitate a comprehensive understanding of cranial anatomy, which is essential for both clinical applications and comparative studies. Also a study on skulls also helps determine species and sex [12]. As research in this field progresses, it is anticipated that further insights will emerge, enhancing our ability to diagnose and treat conditions affecting the cranial region in both sheep and humans, thereby underscoring the significance of this research in advancing medical knowledge and practice.

## 2. Materials and Methods

### 2.1. Collection of the Specimens

The heads of three male lambs (3‐month‐old, 30 ± 0.75 kg) and five adult male Romane sheep (4 years old, 71.5 ± 1.22 kg) were included in the study. Samples were chosen from slaughtered sheep from one of the farms around Tehran, Iran. The samples were collected on the same day, and CT scans were performed fresh and unfrozen. Heads were transferred to the faculty of veterinary medicine at the University of Tehran for CT scan imaging. All procedures were conducted in accordance with approved animal welfare guidelines affirmed by the ethics committee of Tehran Veterinary Medicine University.

### 2.2. CT Imaging

A Somatom Spirit 2 CT machine, a third‐generation spiral CT scanner with two rows of detectors, manufactured by Siemens, was utilized for this study. The CT imaging parameters included a tube voltage of 120 kVp, a tube current of 70 mAs, and a slice thickness of 1.0 mm.

### 2.3. 2D and 3D Study

**FIGURE 1:**
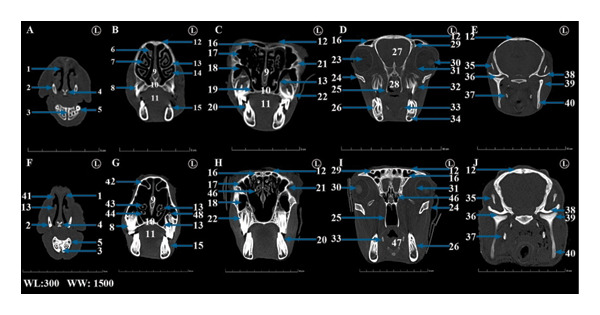
2D transverse CT scan images (bone window) in the Romane lamb (A‐E) and skeletally mature sheep (F‐J). (A&F) At the level of diastema; (B&G) at the level of 1st mandibular premolar; (C&H) at the level of 1st maxillary and 2nd mandibular molar teeth; (D&I) at the level of the mid part of the eyeball; (E&J) at the level of TMJ. (1) Dorsal nasal meatus; (2) nasal process of incisive bone; (3) mandibular symphysis; (4) palatine plexus; (5) 4th left incisive tooth; (6) dorsal conchal sinus; (7) sinus of dorsal spiral lamella of ventral nasal concha; (8) 1st maxillary premolar tooth; (9) nasal septum; (10) hard palate; (11) tongue; (12) frontal bone; (13) ventral nasal concha; (14) sinus of ventral spiral lamella of ventral nasal concha; (15) 1st mandibular premolar tooth; (16) frontal sinus; (17) lacrimal sinus; (18) maxillary sinus; (19) palatine sinus; (20) 2nd mandibular molar teeth; (21) nasolacrimal duct; (22) 1st maxillary molar teeth; (23) eyeball; (24) zygomatic arch; (25) pterygoid hamulus; (26) 3rd mandibular tooth; (27) brain; (28) choanae; (29) supraorbital canal; (30) lens of eye; (31) sclera; (32) 2nd maxillary molar tooth; (33) epihyoid; (34) mandibular canal; (35) coronoid process of mandible; (36) temporomandibular joint; (37) thyrohyoid; (38) 2nd mandibular molar tooth; (38) zygomatic process of temporal bone; (39) condylar process of mandible; (40) mandible; (41) alar fold of ventral nasal concha; (42) dorsal conchal sinus; (43) dorsal spiral lamella of ventral nasal concha; (44) ventral spiral lamella of ventral nasal concha; (46) ethmoidal labyrinth and sinuses; (47) pharynx; (48) infraorbital canal.

**FIGURE 2:**
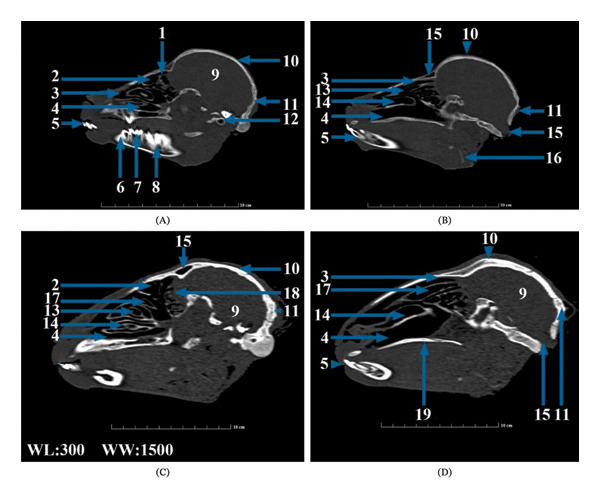
2D sagittal CT scan images (bone window) in the Romane lamb (A&B) and skeletally mature sheep (C&D). (A&C) At the level of the tympanic bulla (B&D), at the level of 2 cm right of the midline section. (1) Dorsal nasal meatus; (2) dorsal conchal sinus; (3) maxillary sinus; (4) palatine sinus; (5) incisive tooth; (6) 1st mandibular premolar tooth; (7) 2nd mandibular premolar tooth; (8) 3rd mandibular premolar tooth maxillary bone; (9) brain; (10) frontal bone; (11) occipital bone; (12) tympanic bullae; (13) sinus of dorsal spiral lamella of ventral nasal concha; (14) sinus of ventral spiral lamella of ventral nasal concha; (15) foramen magnum; (16) ceratohyoid; (17) middle conchal sinus; (18) ethmoidal labyrinth.

**FIGURE 3:**
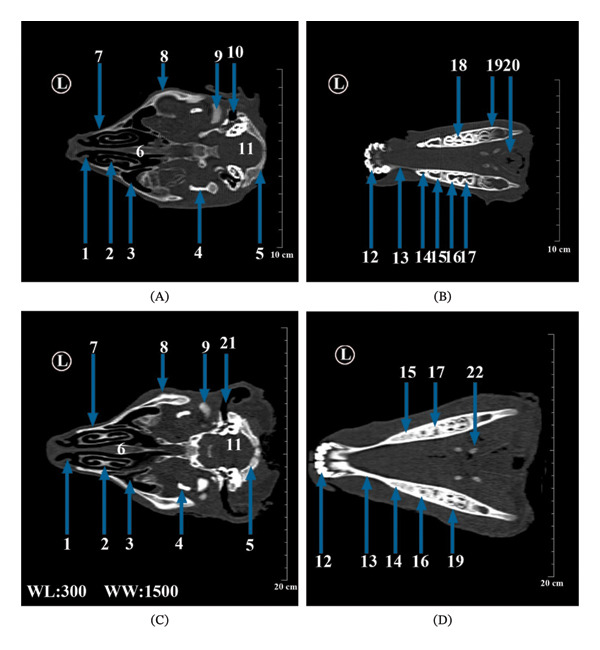
2D sagittal CT scan images (bone window) in the Romane lamb (A&B) and skeletally mature sheep(C&D). (A&C) At the level of the tympanic bulla (B&D), at the level of the mandibular teeth. (1) Alar fold of ventral nasal concha; (2) ventral nasal concha; (3) maxillary sinus; (4) ramous of mandible; (5) occipital bone; (6) nasal septum; (7) nasal bone; (8) maxillary bone; (9) condylar process of mandible; (10) tympanic bullae; (11) brain; (12) incisive tooth; (13) diastema; (14) 1st mandibular premolar tooth; (15) 2nd mandibular premolar tooth; (16) 3rd mandibular premolar tooth; (17) 1st mandibular molar tooth; (18) 2nd mandibular molar tooth; (19) 3rd mandibular molar tooth; (20) pharynx; (21) external ear canal; (22) stylohyoid.

**FIGURE 4:**
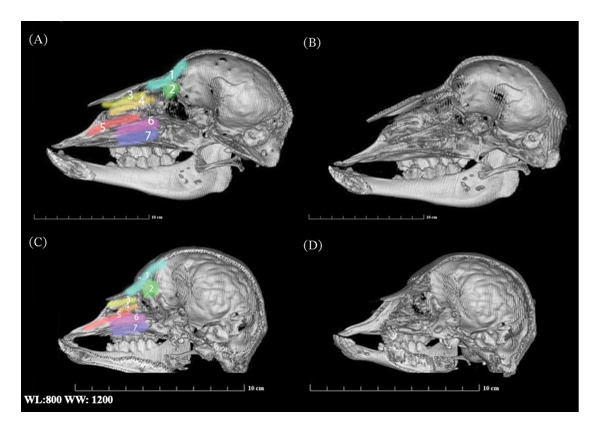
Sagittal section of the 3D reconstructed CT scan (osseous shaped‐vp) of the skulls of the skeletally mature (A and B) and lamb (C and D) Romane sheep showing location of nasals and paranasal sinuses in related to tooth. The light blue (1) color indicates location of frontal sinus, green (2) is for lacrimal sinus, yellow (3), orange (4) and red (5) one is related to dorsal, middle and ventral conchal sinuses, respectively and purple (6) and blue (7) color are showing maxillary and palatine sinuses.

Transverse, sagittal, and dorsal CT scan images of lambs and skeletally mature sheep of the Romane breed were examined using Radiant version 2025.1 software. Also, 3D images were analyzed using the Marcopacs division v14048.14 software. 2D CT images, including transverse, sagittal, and dorsal sections, are shown in Figures 1–3. The location of nasals and paranasal sinuses in relation to the teeth is shown in Figure 4 and Table 1.

**TABLE 1:** The location of nasal and paranasal sinuses of lamb and skeletally mature Romane sheep in relation to the teeth.

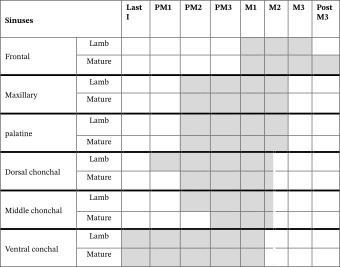

*Note:* The gray color indicates the presence of structure. The cells of the table that are half gray mean that there is the presence of structure up to a part of this section.

### 2.4. Morphometric and Volumetric Study

**FIGURE 5:**
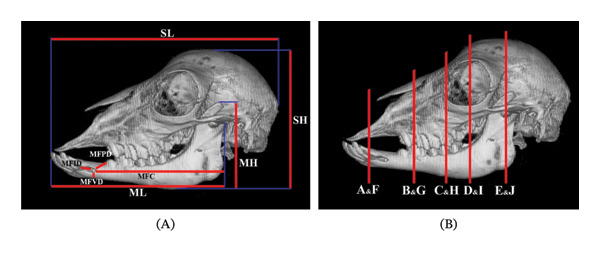
3D reconstruction (osseous shaped‐vp) of the skull of a Romane skeletally mature sheep in lateral view. (A) Morphometric parameters. *Abbreviations:* MFC, distance from the caudal margin of the mandibular ramus to the mental foramen; MFID, distance from the mental foramen to the most lateral incisive tooth; MFPD, distance from the mental foramen to the cranial margin of the first premolar tooth; MFVD, distance from the mental foramen to the ventral margin of the mandibular body; MH, mandibular height; ML, mandibular length; SH, skull height; SL, skull length. (B) Section location of Figure 1.

The morphometry of the skull of Romane sheep was studied using the RadiAnt application. The parameters used in this study were taken from “CT anatomy of the head in the Ile de France sheep” [3] and are shown in Figure 5. Skull parameters were measured and are shown in Tables 2–5.

**TABLE 2:** Morphometric results of head (unit of measurement is cm) in the skull of Romane sheep.

No	SH	SL	SW	NL	ED	NW
L1	7.96	13.58	7.36	7.52	2.32	1.59
L2	7.69	14.15	7.25	7.66	2.25	1.69
L3	7.77	13.69	7.15	7.50	2.15	1.42
Mean ± SD	7.80 ± 0.13	13.80 ± 0.30	7.25 ± 0.10	7.56 ± 0.08	2.24 ± 0.08	1.56 ± 0.13
M1	13.83	23.34	11.79	14.58	4.19	3.43
M2	14.22	23.21	11.44	14.22	4.43	2.93
M3	13.43	22.94	12.09	14.22	3.91	3.55
Mean ± SD	13.82 ± 0.39	23.16 ± 0.20	11.77 ± 0.32	14.34 ± 0.20	4.17 ± 0.26	3.30 ± 0.32

*Note:* NL, nasal cavity length; NW, nasal cavity width.

Abbreviations: ED, eyeball diameter; SH, skull height; SL, skull length; SW, skull width.

**TABLE 3:** Morphometric results of mandible (unit of measurement is cm) in the skull of Romane sheep.

No	MH	ML	MFID	MFPD	MFVD	MFC
L1	4.10	9.84	1.12	0.88	0.56	7.25
L2	4.44	9.68	1.02	0.89	0.49	7.77
L3	3.89	7.88	1.15	0.77	0.65	7.15
Mean ± SD	4.14 ± 0.27	9.13 ± 1.08	1.09 ± 0.06	0.84 ± 0.06	0.56 ± 0.08	7.39 ± 0.33
M1	8.86	18.06	2.02	1.53	0.98	12.26
M2	8.66	18.12	2.31	1.23	1.08	12.11
M3	9.06	18.26	2.33	1.71	0.95	12.22
Mean ± SD	8.86 ± 0.20	18.14 ± 0.10	2.22 ± 0.17	1.49 ± 0.24	1.00 ± 0.06	12.19 ± 0.07

*Note:* MFC, distance from the caudal margin of the mandibular ramus to the mental foramen; MFID, distance from the mental foramen to the most lateral incisive tooth; MFPD, distance from the mental foramen to the cranial margin of the first premolar tooth; MFVD, distance from the mental foramen to the ventral margin of the mandibular body.

Abbreviations: MH, mandibular height; ML, mandibular length.

**TABLE 4:** Morphometric results of hyoid bone (unit of measurement is cm) in the skull of Romane sheep.

No	S	E	C	T
L1	3.12	1.02	1.10	1.15
L2	3.15	0.99	1.12	1.06
L3	3.20	0.89	1.06	1.10
Mean ± SD	3.15 ± 0.04	0.96 ± 0.06	1.09 ± 0.03	1.10 ± 0.04
M1	5.71	1.45	1.68	2.07
M2	5.51	1.55	1.71	2.15
M3	8.01	1.66	1.71	2.17
Mean ± SD	6.41 ± 1.38	1.55 ± 0.10	1.70 ± 0.01	2.13 ± 0.05

*Note:* C, ceratohyoid length; E, epihyoid length; S, stylohyoid length; T, thyrohyoid length.

**TABLE 5:** Morphometric results of teeth (unit of measurement is cm) in the skull of Romane sheep. First incisor length (I1), 1st premolar length (PM1), 2nd premolar length (PM2), 3rd premolar length (PM3), 1st molar length (M1), 2nd molar length (M2), 3rd molar length (M3).

No	I_1_	PM_1_	PM_2_	PM_3_	M_1_	M_2_	M_3_
L1	0.26	0.89	0.98	1.50	1.44	1.69	1.54
L2	0.33	0.78	1.03	1.55	1.36	1.77	1.50
L3	0.29	0.77	0.97	1.56	1.39	1.59	1.55
Mean ± SD	0.29 ± 0.03	0.81 ± 0.06	0.99 ± 0.03	1.53 ± 0.03	1.39 ± 0.04	1.68 ± 0.09	1.53 ± 0.02
M1	0.49	1.02	1.05	1.59	1.35	1.60	1.65
M2	0.51	1.12	1.31	1.62	1.41	1.66	1.71
M3	0.55	1.21	1.08	1.61	1.51	1.65	1.66
Mean ± SD	0.51 ± 0.03	1.11 ± 0.09	1.14 ± 0.14	1.60 ± 0.01	1.42 ± 0.08	1.63 ± 0.03	1.67 ± 0.03

Volume estimation was performed, consequently utilizing the computer program accessible on the CT scan system (Syngo MMWP VE40A software). For this purpose, the study area was introduced to the software in the bone window, and the software automatically showed the boundaries of the study area in three available views (cross, sagittal, and coronal sections), and if approved, the volume per cm^3^. The volumetric results are shown in Tables 6and 7.

**TABLE 6:** Volumetric results of head (unit of measurement is cm^3^) in the skull of Romane sheep.

No	HV	NCV	OCV	OrCV	BV	TV
L1	321.13	20.22	21.24	10.75	68.67	0.33
L2	335.12	19.80	25.25	11.12	69.25	0.40
L3	366.56	25.50	20.19	12.15	75.13	0.38
Mean ± SD	340.93 ± 23.26	21.84 ± 3.17	22.22 ± 2.67	11.34 ± 0.72	71.01 ± 3.57	0.37 ± 0.03
M1	615.36	39.05	44.62	19.06	118.65	0.62
M2	614.21	37.15	43.21	18.33	117.44	0.55
M3	617.36	40.11	45.12	20.46	119.32	0.91
Mean ± SD	615 ± 1.59	38.77 ± 1.49	44.31 ± 0.90	19.28 ± 1.08	118.47 ± 0.95	0.69 ± 0.19

*Note:* OrCV, orbital cavity volume in the left half of the skull; TV, tympanic bullae volume in the left half of the skull.

Abbreviations: BV, brain volume; HV, head volume; NCV, nasal cavity volume; OCV, oral cavity volume.

**TABLE 7:** Volumetric results of sinuses (unit of measurement is cm^3^) in the skull of Romane sheep.

No	Frontal sinus	Maxillary sinus	Palatine sinus	Dorsal conchal sinus	Middle conchal sinus	Ventral conchal sinus
L1	10.33	3.07	0.64	0.52	0.32	0.62
L2	11.25	3.25	0.55	0.44	0.35	0.59
L3	12.22	3.15	0.78	0.55	0.40	0.64
Mean ± SD	11.26 ± 0.94	3.15 ± 0.90	0.65 ± 0.11	0.50 ± 0.05	0.35 ± 0.04	0.61 ± 0.02
M1	18.25	5.15	1.12	0.89	0.62	0.77
M2	18.33	5.32	1.22	0.91	0.66	0.73
M3	18.15	5.25	1.23	0.94	0.63	0.79
Mean ± SD	18.24 ± 0.09	5.24 ± 0.08	1.19 ± 0.06	0.91 ± 0.02	0.63 ± 0.02	0.76 ± 0.03

*Note:* The volume of the frontal sinus in the right and left half of the skull and also the maxillary, palatine, dorsal, and middle conchal sinus in the left half of the skull.

## 3. Results

The CT examination of the skull in a Romane sheep is presented in three parts. In the first part, which is related to the examination of 2D and 3D images, the images in each slice are explained in detail. In the second part, which is related to the morphometric results, the measurements of different parts of the skull, lower jaw, laminar apparatus, and teeth are included. In the third part, the results of the volume measurement of the skull and its sinuses are described.

### 3.1. 2D and 3D CT Scan

In this part, CT scan sections of the skull of a lamb and skeletally mature Romane sheep are located in 5 parts, which contain cranial bones, facial bones, sinuses, mandible, teeth, and hyoid apparatus based on different parts of the head.

#### 3.1.1. Cranial Bones

Cranial bones are formed by a set of bones that ultimately protect the brain. These bones include the frontal, parietal, temporal, occipital, and sphenoid bones, from the rostral aspect of the skull to the caudal, collectively protecting and separating the brain and other related tissues from the rest of the head structures.

The frontal bone, which is the largest bone of the skull in Romane sheep, forms the roof of the skull and is connected to the facial bones in the rostral aspect and to the parietal bone in the caudal aspect (Figures 1 and 2). It is also connected to the temporal bones on either side (Figure 1).

The frontal bone in the lamb and skeletally mature Romane sheep had the supraorbital canal in the middle. This canal was responsible for connecting the supraorbital foramen and the orbit (Figure 1D,I). This canal in lambs was shorter and had a more vertical angle than in skeletally mature sheep, due to the smaller skull.

The temporal bone is located on the lateral aspect of the skull and articulates with the mandible, eyes, and the lateral edge of the brain. The parietal bone is also located between the frontal and occipital bones, which in the Romane lamb had a clear border with the bones as mentioned above due to the incomplete ossification of the bone sutures.

The occipital bone was the caudal bone of the skull in the Romane sheep. It consists of several symmetrical parts, including condyles and a paracondylar process. This bone also has a foramen magnum, which is the exit point of the spinal cord (Figure 2). The sphenoid bone forms the rostral part of the base of the cranium.

The tympanic bullae contain two single air‐filled compartments surrounded by bone and communicate with the external ear canal (Figure 3A,C).

#### 3.1.2. Facial Bones

The structures forming the facial bones include the bones that lie in the rostral part of the cranium and form cavities such as the nasal and oral cavities. In the Romane sheep, these bones included the nasal bone, incisive, maxilla, zygomatic, and lacrimal.

In lamb and skeletally mature Romane sheep, the nasal bone was located in the rostral aspect of the frontal bone and forms the roof of the nasal cavity (Figure 2). The lateral wall of the nasal cavity (formed primarily by the maxilla and incisive bone) was created by the incisive and maxillary bones (Figure 3A,C). The nasal process of the incisive bone in lamb and skeletally mature Romane sheep was formed by the nasal process of the incisive bone (Figure 1A,F).

Nasal conchae and nasal meatuses were located in the rostral aspect of the nasal cavity. The nasal conchae in this breed included dorsal, middle, and ventral nasal conchae (from dorsal to ventral aspect of the nasal cavity). Also, these conchae continued until the ethmoidal labyrinth (Figures 1H,I and 2A,C). The nasal cavity is divided into right and left parts by the nasal septum by nasal septum (Figure 1).

**FIGURE 6:**
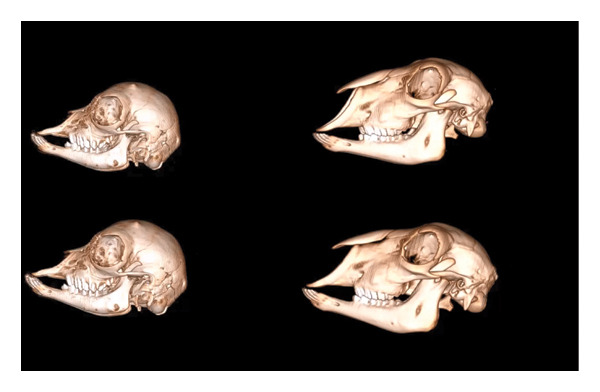
3D reconstructed images of CT scan of the skulls of skeletally mature (right) and lamb (left) Romane sheep. The full video can be accessed in the supporting information.

The dorsal nasal concha in the Romane lamb starts at the level of the first mandibular premolar tooth and ends at the level of the mid part of the mandibular second molar (M2) tooth. But in skeletally mature Romane sheep, it started at the level of the mandibular second premolar tooth and continued to the mid part of the mandibular second molar tooth (Figures 1 and 4). The middle nasal concha was from the level of the mandibular second premolar tooth in the lamb and the mandibular third premolar tooth in the skeletally mature Romane sheep. In both lamb and skeletally mature Romane sheep, middle conchae end at the level of the mid part of the mandibular second molar tooth (Figures 2 and 4). The ventral nasal concha was ventral nasal concha and divided into dorsal and ventral parts. Ventral nasal concha was started at the left of the caudal aspect of the last incisive tooth to the first mandibular molar tooth in both lamb and skeletally mature sheep (Figures 1, 3, and 4). In clip 1, different views of the skull structure in adult and immature Romane sheep are shown in a comparative manner(Figure 6).

#### 3.1.3. Sinuses

The sinuses in the skull of the Romane sheep are located in two groups: paranasal and nasal sinuses. In lamb and skeletally mature Romane sheep, all the frontal, maxillary, palatine, and lacrimal sinuses are present. Also, there are all of the nasal sinuses, which contain the dorsal, middle, and ventral conchal sinus are, present in this small ruminant breed.

The frontal sinus, which is the largest sinus in the skull of the Romane sheep, starts from the mandibular first molar tooth and continues to the last mandibular molar tooth in lambs (Figures 1C,D and 2B). But in older animals, this beginning and end are different, and it is longer than in lambs and starts from the first mandibular molar tooth and continues to 1 cm caudal to the last mandibular molar tooth (Figures 1H,I and 2C). Also, in the middle part of this sinus, there was the supraorbital canal, which connected the supraorbital foramen and the orbit (Figure 1D,I).

The lacrimal sinus was part of the frontal sinus and was located on the sides of this sinus. In both lamb and mature sheep, this sinus was located at the anterior edge of the orbit (Figure 1C, H).

The maxillary and palatine sinuses both start at approximately the same point (from the secondary maxillary premolar tooth) and continue parallel to the maxillary teeth to the secondary maxillary molar tooth (Figures 1–3) in both lamb and mature sheep.

The infraorbital canal, which was the largest intracranial canal in this breed, was located in the upper part of the roots of the maxillary teeth and in lamb it started from mid part of second premolar (P2) tooth and continued to rostroventral aspect of orbit, but in skeletally mature Romane sheep it started from first maxillary premolar tooth and continued to rostroventral aspect of orbit (Figure 1G). There was also a small canal between the ventral part of the nasal cavity and the rostrodorsal aspect of the orbit, which was in the area of the second and third maxillary premolar tooth (Figure 1C,H).

Nasal sinuses contain the dorsal, middle, and ventral conchal sinuses. The dorsal conchal sinus was in the dorsal aspect of the nasal cavity (Figure 1B,G). The middle conchal sinus was between the dorsal and ventral ones (Figure 2C,D). The ventral conchal sinus was divided into dorsal and ventral compartments and located in the ventral aspect of the nasal cavity (Figures 1B and 2C,D).

#### 3.1.4. Mandible

The mandible was a two‐part bone that formed a symphysis at the rostral part (Figure 1A,F), with incisors in the rostral part, a toothless section (diastema) in the middle, and premolars and molars in the caudal part. This bone had different sections at the caudal aspect, including the mandibular ramus, coronoid process of the mandible, and condylar process of the mandible (Figure 1E,J). The temporomandibular joint (TMJ) is also between the condylar process of the mandible and the zygomatic process of the temporal bone, which causes mandibular movements.

There is a large canal in the mid part of the mandibular body, which begins on the medial surface of the ramus of the mandible, at the mandibular foramen, and runs obliquely downward and forward in the ramus; then forward horizontally in the body until the mental foramen.

#### 3.1.5. Teeth

The dental formula in Romane sheep was 4/4, 0/0, 3/3, 3/3. The maxilla and mandible lacked canines, and the canine location was toothless (diastema). There are four incisors present in each half of the mandible, while the maxilla lacks incisors. Also, there were 3 premolars and molars in each half of the jaw in lambs and adult sheep (Figures 1, 2A, and 3B,D). It is worth noting that the last mandibular molar in one of the lambs, which was slightly younger than the others in terms of age, had not fully erupted (Figure 1D).

### 3.2. Morphometric Results

The results of the morphometric studies of the skull in lamb and skeletally mature Romane sheep are presented in the form of 4 tables titled Morphometric results of head (Table 2), mandible (Table 3), hyoid bone (Table 4), and teeth (Table 5), some of which are briefly presented below.

The skull measurements of Romane lamb show an average height of 7.80 cm, length of 13.80 cm, and width of 7.25 cm. In skeletally mature Romane sheep, these dimensions are larger, with a skull length of 23.16 cm, height of 13.82 cm, and width of 11.77 cm. Overall, mature sheep have significantly larger skull dimensions compared to lambs.

In Romane lambs, the mandible has an average height of 4.14 cm and a length of 9.13 cm. Skeletally mature Romane sheep show larger dimensions, with a mandibular height of 8.86 cm and a length of 18.14 cm. The hyoid bone components in mature sheep are approximately twice as long as those in lambs.

Teeth measurements in Romane lamb show the lengths of incisors averaging 0.29 cm, premolars ranging from 0.81 cm to 1.53 cm, and molars ranging from 1.39 cm to 1.53 cm. In skeletally mature Romane sheep, these measurements are higher, with incisors averaging 0.51 cm, premolars 1.60 cm, and molars up to 1.67 cm.

### 3.3. Volumetric Results

The results of the skull volumetric studies in lamb and skeletally mature Romane sheep are presented in the form of two tables titled skull volume (Table 6) and nasal and paranasal sinuses volume (Table 7), some of which are briefly presented below.

The head volume of Romane lamb averages 340.93 cm^3^, with the nasal cavity volume at 21.84 cm^3^, oral cavity volume at 22.22 cm^3^, orbital cavity volume at 11.34 cm^3^, brain volume at 71.01 cm^3^, and tympanic bullae volume at 0.37 cm^3^. Skeletally mature Romane sheep have significantly larger head volumes, averaging 615 cm^3^, with other cavities proportionally increasing. Mature sheep have nearly double the head and cavity volumes compared to lambs, demonstrating substantial cranial development.

In the Romane lamb, sinus volumes include an average frontal sinus volume of 11.26 cm^3^, maxillary sinus of 3.15 cm^3^, palatine sinus of 0.65 cm^3^, dorsal conchal sinus of 0.50 cm^3^, and middle conchal sinus of 0.35 cm^3^. In skeletally mature Romane sheep, these volumes increase, with the frontal sinus reaching 18.24 cm^3^ and the maxillary sinus 5.24 cm^3^. Sinus volumes in mature sheep are significantly larger than in lambs, highlighting extensive sinus development with age.

The ratio of the volume of the oral cavity and nasal cavity to the volume of the head was 6% for both cavities in skeletally mature sheep. The brain also occupies approximately 20% of the head volume. The eyes also accounted for a total of 6% of the head volume. The ratio of the largest paranasal sinus (frontal sinus) to head volume was approximately 0.02, and the ratio of the largest nasal sinus (ventral) to nasal cavity volume was approximately 0.02. Also, the ratio of the maxillary and palatine sinuses (on both sides) to the frontal sinus was 0.56 and 0.13, respectively. In the present study on lambs, the oral cavity and nasal cavity each accounted for 6% of the total head volume. The brain occupied approximately 20% of the head volume, while the combined volume of the eyes represented 6% of the head volume. Among the paranasal sinuses, the frontal sinus was the largest, with a volume ratio of 0.03 relative to the total head volume. Within the nasal cavity, the ventral nasal sinus also exhibited a volume ratio of 0.03 relative to the nasal cavity volume. Furthermore, the combined volume of the maxillary and palatine sinuses (bilaterally) corresponded to 0.54 and 0.12 times that of the frontal sinus, respectively.

## 4. Discussion

The Romane sheep, a breed known for its distinctive characteristics, exhibits notable variations in skull anatomy that reflect both evolutionary adaptations and breed‐specific traits. The skull of the Romane sheep, like that of other sheep breeds, has undergone morphological changes due to domestication, which can be traced through archaeological findings that highlight alterations in cranium and horn structures over time [13]. These changes are significant as they not only indicate the breed’s adaptation to domestication but also provide insights into the genetic relationships among different sheep breeds. For instance, studies have shown that skull morphology can serve as a reliable indicator for distinguishing between breeds, with specific measurements such as skull indices being crucial for classification [14]. Furthermore, the Romane sheep’s skull shares similarities with the human skull in terms of bone thickness and anatomical features, making it a valuable model for various medical and veterinary studies [7, 15].

In this study, which investigated the anatomical characteristics of Romane sheep, a comparison was made for the first time between lamb and skeletally mature sheep in morphometric and volumetric studies. After analyzing the obtained CT data and comparing lamb and mature sheep, no significant anatomical differences were observed at different ages in this breed. The significant difference between the two age groups was in the morphometry and volume of various skull structures.

Infra and supraorbital canals, which connect the eye with the outside of the skull, and the nasolacrimal canal, which connects the inner canthus of the eye and the nasal cavity, have been reported in all small ruminant species such as Urial, Ile de France sheep, Alborz wild sheep, Shal sheep, and Saanen goat, and also in Rhesus monkey [16]. The exact location of the infraorbital foramen in lamb and skeletally mature Romane sheep was at the level of the second maxillary premolar tooth, which was the same as that of Shal and Ile de France sheep, and contrary to the results of Urial and Alborz wild sheep studies at the level of the third premolar tooth [3, 5, 10, 17].

The frontal, maxillary, and lacrimal sinuses are present in all species studied so far. However, the palatine sinus is present in all ruminant species except Egyptian native sheep, Saanen goat, and One‐Humped Camel, as in the Romane sheep [6, 17, 18]. The sphenoid sinus has been reported in some small ruminant breeds such as Urial, Ile de France, and Alborz wild sheep [3–5], but was not observed in Romane sheep breeds such as Shal sheep and Rayini goat [10, 19]. A comparison of the nasal and paranasal sinuses in the species and breeds studied so far is summarized in Table 8.

**TABLE 8:** Comparison of nasal and paranasal sinuses in different ruminant species and breeds.

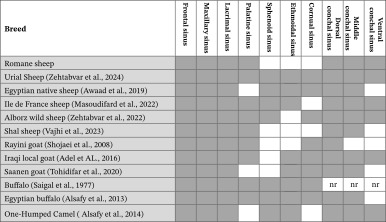

*Note:* The gray color indicates the presence of structure.

Abbreviation: nr, not reported.

Among the nasal sinuses, the dorsal conchal sinus has been reported in all the studied species. The middle conchal sinus was absent only in the Rayini goat [19]. Ventral conchal sinus has been reported in some species, such as Ile de France and Urial sheep [3, 5], which is similar to our results, but it has not been observed in the Shal and Rayini sheep and Egyptian buffalo [10, 19, 20].

The skull length and width of skeletally mature Romane sheep were smaller compared to those of Urial, Ile de France sheep, and Alborz wild sheep. But its height was more than these two, the Ile de France and Alborz wild sheep [3, 4]. The length of the nasal cavity in Alborz wild sheep is reported to be 15.02 ± 0.74 cm, which is larger than that of Romane sheep, but the nasal cavity length in Urial sheep was smaller than that of this breed [3, 4]. The diameter of the eyeball in Romane sheep was larger than that of Urial sheep and smaller compared to Ile de France and Alborz wild sheep [3–5].

Comparing the length and height of the mandible in skeletally mature Romane sheep with Urial, Ile de France, and Alborz wild sheep indicates that the mandible is smaller in this breed compared to the mentioned sheep [3–5]. Sex differences have also been noted in the morphometric and volumetric characteristics of Romanov sheep and porcupine [12, 21].

The dental formula in Romane sheep was similar to that of other small ruminants. The sizes of the smallest and largest tooth in this breed were 0.51 ± 0.03 and 1.67 ± 0.03 cm, respectively, corresponding to the incisor tooth and the last molar tooth. In Ile de France sheep, this value is reported as 0.58 ± 0.01 and 1.63 ± 0.13 cm in males, respectively. It is also 0.67 ± 0.33 and 2.05 ± 0.39 cm in Alborz wild sheep. The smallest and largest teeth in the two mentioned breeds are incisors and last molars, respectively [3, 4].

The volume of the head in Romane sheep was less compared to that of Ile de France sheep, which was 2770 ± 90.00 cm^3^, and compared to the volume of 1636.33 ± 73.34 cm^3^ of Alborz wild sheep [3, 4]. The ratio of nasal cavity and oral cavity to head volume was reported as 0.1 and 0.07 in Ile de France sheep and 0.1 and 0.09 in Alborz wild sheep, respectively. However, these ratios were lower in Romane sheep. Also, the ratio of the brain to the head volume was 0.04 in Ile de France sheep and 0.24 in Alborz wild sheep, which, compared to Romane, was higher in Alborz and lower in Ile de France sheep [3, 4].

In the present study, the ratios of the oral and nasal cavity volumes to the total head volume in immature sheep were both 0.06, which were comparable to the values reported for skeletally mature sheep. This similarity suggests that despite the overall enlargement of the skull and increase in head size during maturation, the relative contribution of the spaces associated with the upper digestive and respiratory tracts remains relatively stable throughout growth. The brain occupied approximately 20% of the total head volume in lambs, which was slightly lower than the value reported in mature sheep (approximately 21%), possibly reflecting differential growth patterns among cranial components, with greater postnatal expansion of bony structures and paranasal sinuses. Similarly, the combined volume of the eyes represented approximately 6% of the head volume in both age groups, indicating a relatively conserved proportional relationship of the orbital structures during development. Among the paranasal sinuses, the frontal sinus was the largest in both immature and mature sheep; however, its ratio to the total head volume was slightly higher in lambs (0.03) than in mature animals (0.02). This difference may indicate relatively rapid development of cranial sinuses during early postnatal growth or variations in the pattern of cranial pneumatization. Furthermore, the ratios of the bilateral maxillary and palatine sinuses to the frontal sinus in lambs (0.54 and 0.12, respectively) were highly similar to those reported in mature sheep (0.56 and 0.13), suggesting that the proportional relationships among the major paranasal sinuses are established early and remain relatively consistent during maturation.

Nevertheless, the small sample size in the present study represents a limitation that precluded statistical evaluation of these ratios. Therefore, studies involving larger cohorts and statistical analyses are necessary to validate these findings and provide more accurate comparisons between mature and immature animals.

In conclusion, the study of the cranial CT anatomy of the Romane sheep, one of the most prominent and internationally recognized breeds, underscores its significant importance in veterinary and genetic research. The Romanov sheep is renowned for its exceptional genetic traits, including high fertility, adaptability to diverse environmental conditions, and robust health, making it a valuable asset in improving sheep flocks worldwide. Establishing a comprehensive understanding of the normal cranial anatomy of this breed provides essential insights into its anatomical and functional structures, which are crucial for identifying abnormalities, diagnosing diseases, and guiding surgical procedures in veterinary practice. Furthermore, these findings serve as a foundational reference for comparative studies across other breeds, aiding in the identification of breed‐specific variations and their implications for breeding programs. The integration of such anatomical data into genetic and breeding strategies can also enhance efforts to preserve genetic diversity and improve overall productivity and resilience in sheep populations. This research highlights the vital role of detailed anatomical studies in advancing both scientific knowledge and practical applications in veterinary medicine and animal husbandry.

## Author Contributions

Omid Zehtabvar: methodology, project administration, supervision, and writing–review and editing. Javad Abbasi: conceptualization, data curation, software, writing–original draft preparation. Majid Masoudifard: funding acquisition and writing. Fateme Pariz: funding acquisition, validation, and writing–review and editing. Javid Sepahtash: funding acquisition, validation, and writing–review and editing. Seyyed Hossein Modarres Tonekabony: formal analysis, resources, software, writing–original draft preparation, and writing–review and editing.

## Funding

No funding was received for this manuscript.

## Ethics Statement

This study was a doctor of veterinary medicine thesis, and all experimental procedures were approved by the Faculty of Veterinary Medicine, University of Tehran (ID: 30704/6/17).

## Conflicts of Interest

The authors declare no conflicts of interest.

## Supporting Information

Additional supporting information can be found online in the Supporting Information section.

## Supporting information


**Supporting Information** The full video can be accessed in the Supporting Information.

## Data Availability

Data are available on request from the authors.
